# The longitudinal associations between ambient air pollution exposure and dementia in the UK: results from the cognitive function and ageing study II and Wales

**DOI:** 10.1186/s12889-024-18723-3

**Published:** 2024-05-04

**Authors:** Yu-Tzu Wu, Nutthida Kitwiroon, Sean Beevers, Benjamin Barratt, Carol Brayne, Ester Cerin, Rachel Franklin, Vikki Houlden, Bob Woods, Eman Zied Abozied, Matthew Prina, Fiona Matthews

**Affiliations:** 1 Population Health Sciences Institute, Faculty of Medical Sciences, Campus for Ageing and Vitality, Newcastle University, Newcastle Upon Tyne, NE4 5PL UK; 2 MRC Centre for Environment and Health, School of Public Health, Imperial College London, London, UK; 3 Cambridge Public Health, University of Cambridge, Cambridge, UK; 4 Mary MacKillop Institute for Health Research, Australian Catholic University, Melbourne, VIC Australia; 5 Centre for Urban and Regional Development Studies (CURDS), School of Geography, Politics and Sociology, Newcastle University, Newcastle Upon Tyne, UK; 6 School of Geography, University of Leeds, Leeds, UK; 7 Dementia Services Development Centre, Bangor University, Bangor, Gwynedd UK

**Keywords:** Dementia, Air pollution, Environmental risk factors, Public health, Cohort studies

## Abstract

**Background:**

Air pollution has been recognised as a potential risk factor for dementia. Yet recent epidemiological research shows mixed evidence. The aim of this study is to investigate the longitudinal associations between ambient air pollution exposure and dementia in older people across five urban and rural areas in the UK.

**Methods:**

This study was based on two population-based cohort studies of 11329 people aged ≥ 65 in the Cognitive Function and Ageing Study II (2008–2011) and Wales (2011–2013). An algorithmic diagnosis method was used to identify dementia cases. Annual concentrations of four air pollutants (NO_2_, O_3_, PM_10_, PM_2.5_) were modelled for the year 2012 and linked via the participants’ postcodes. Multistate modelling was used to examine the effects of exposure to air pollutants on incident dementia incorporating death and adjusting for sociodemographic factors and area deprivation. A random-effect meta-analysis was carried out to summarise results from the current and nine existing cohort studies.

**Results:**

Higher exposure levels of NO_2_ (HR: 1.04; 95% CI: 0.94, 1.14), O_3_ (HR: 0.90; 95% CI: 0.70, 1.15), PM_10_ (HR: 1.17; 95% CI: 0.86, 1.58), PM_2.5_ (HR: 1.41; 95% CI: 0.71, 2.79) were not strongly associated with dementia in the two UK-based cohorts. Inconsistent directions and strengths of the associations were observed across the two cohorts, five areas, and nine existing studies.

**Conclusions:**

In contrast to the literature, this study did not find clear associations between air pollution and dementia. Future research needs to investigate how methodological and contextual factors can affect evidence in this field and clarity the influence of air pollution exposure on cognitive health over the lifecourse.

**Supplementary Information:**

The online version contains supplementary material available at 10.1186/s12889-024-18723-3.

## Introduction

Dementia has been recognised as a public health priority in ageing societies across the world [1, 2]. To find ways to reduce the risk of dementia, many studies have identified a wide range of risk factors at the individual level, such as low education attainment, diabetes, and hypertension [3, 4]. In recent years, several studies have started to investigate environmental factors related to cognitive health [5, 6]. A large number of studies have focused on ambient (outdoor) air pollution, which has a detrimental impact on cardiovascular diseases and inflammation and may also have a negative influence on brain health in older age [7–11].

In the Lancet 2020 commission report on *Dementia prevention, intervention, and care* [12], air pollution has been listed as one of the 12 key risk factors in later life and is estimated to account for 2.3% of worldwide dementia cases [12]. Yet this estimate was based on results from one study, using a health administrative database of over two million adults in Ontario, Canada [13]. This large population-based study has advantages of a long follow-up period, with a diverse study population registered in publicly funded healthcare system. While this study provided high-quality estimates at the time of the commission report preparation, evidence from other studies were not incorporated. In systematic reviews, specific air pollutants, including PM_2.5_, NO_2_, NO_x_, O_3_, and CO, have been shown to be related to increased risk of dementia in individual studies [5, 11]. However, recent meta-analyses [6–8] showed mixed evidence, with reported a detrimental impact of PM_2.5_ on dementia but unclear associations for NO_2_, NO_x_, and O_3_. The effects also appear to vary across studies [7, 8]. With different timing and methods of exposure and outcome measurements in the existing studies, it is difficult to suggest how air pollution can influence brain health in older populations across diverse settings.

In the UK, the evidence on air pollution and dementia is mainly based on data from the Clinical Practice Research Datalink (CPRD; primary care database) in the Greater London area [14], the UK Biobank (a healthy volunteer cohort) [15], and the English Longitudinal Study of Ageing (ELSA; a population-derived cohort study) [16]. While results from CPRD and UK Biobank suggested detrimental impacts of PM_2.5_ and NO_2_ on incident dementia [14, 15], ELSA reported no associations for these air pollutants [16]. The three studies used medical records or self-reported diagnosis to identify dementia cases and are, therefore, susceptible to diverse factors that could affect case ascertainment, including help-seeking behaviour of individuals, availability of health services, and variation in assessment methods across clinicians, regions, and time [17, 18]. These issues can be coupled with disadvantaged socioeconomic backgrounds and poor living conditions, which are closely related to air pollution exposure and may mask its true effect on dementia. To address these potential limitations, it is essential to investigate the impacts of air pollution exposure on dementia in epidemiological cohort studies that apply the same diagnostic methods across participants from diverse backgrounds and settings.

Using two multicentre population-based cohort studies of older people in the UK, the aim of this study is to investigate the longitudinal associations between air pollution exposure and dementia, while incorporating death, a competing risk outcome in older age. The analysis investigated the associations in the two cohort studies and the five urban and rural areas. To synthesise evidence in this field, the analysis further incorporated results from the existing longitudinal cohort studies reporting the associations.

## Methods

### Study population

This study was based on the Cognitive Function and Ageing Study (CFAS) II and Wales, two population-based cohort studies of people aged 65 or above in England and Wales, UK. CFAS II (www.cfas.ac.uk/cfas-ii/) included participants from three areas in England, including two urban sites (Newcastle upon Tyne and Nottingham) and one rural site (Cambridgeshire). CFAS Wales (cfaswales.bangor.ac.uk) had two sites in rural (Gwynedd and Ynys Môn) and urban (Neath Port Talbot) areas of Wales. The two studies were approved by local research ethics committees in accordance with the principles of the Declaration of Helsinki. CFAS II was approved by Cambridgeshire 4 Research Ethics Committee (reference 07/MRE05/48) and full information is provided on the study website. CFAS Wales was approved by the North Wales Research Ethics Committee (West) (reference 10/WNo01/37). Informed consent was obtained from all the participants.

The two studies had the same study design, which is described in detail elsewhere [19–21]. In brief, the study population was sampled from primary care registrations to identify eligible participants in the study areas and an introductory letter was sent by local General Practitioners. For each centre, participants were recruited with equal numbers of those aged 65–74 years and ≥ 75 years. Trained interviewers visited the participants and carried out standardised computerised questionnaires on sociodemographic information, health conditions, lifestyle, and medication. Tests for cognitive function and psychiatric assessments were embedded in the questionnaires. The baseline study population included 7762 people in CFAS II (2008–2011) and 3593 in CFAS Wales (2011–2013). A two-year follow-up was carried out for CFAS II (2011–2013) and Wales (2013–2016). This study excluded a small number of participants who had unavailable data on baseline dementia diagnosis (9 in CFAS II and 17 in CFAS Wales). This left 7753 in CFAS II and 3576 in CFAS Wales for analysis.

### Outcome variables: dementia and death

The Geriatric Mental State examination (GMS) and its computerised algorithmic programme, Automated Geriatric Examination for Computer Assisted Taxonomy (AGECAT) [22], was used to identify dementia cases in the baseline and follow-up waves of CFAS II and Wales. The GMS-AGECAT has been validated to provide a dementia diagnosis according to the Diagnostic and Statistical Manual of Mental Disorders, revised third edition (DSM-III-R) [23]. More detailed information on dementia diagnosis in CFAS is provided in previous publications [19, 20].

Information on death was identified through data linkage to national death certification. The months and years of death were obtained until the end of 2015 were available for CFAS II and updated until February 2019 for CFAS Wales. A censored date was set at 31st December 2015 for CFAS II and 1st March 2019 for CFAS Wales.

### Air pollution exposure

Four air pollutants, including nitrogen dioxide (NO_2_), ozone (O_3_), and particulate matter with a diameter ≤ 10 microns (PM_10_) and ≤ 2.5 microns (PM_2.5_), were modelled for the study areas of CFAS II and Wales. The Community Multiscale Air Quality Urban (CMAQ) model coupled with a local road scale model hereafter called CMAQ-Urban model was used to estimate hourly air pollution levels at a resolution of 20 × 20 m [24]. The CMAQ-Urban model incorporates meteorological fields from the Weather Research and Forecasting Model (WRF) [25], anthropogenic emissions from the UK National Atmospheric Emissions Inventory (NAEI), and the Imperial College road emissions model, natural emissions from the Biogenic Emission Inventory System version 3 (BEIS3) [26] and evaluated using the measurements from the London Air Quality Monitoring Network (LAQN) and the UK Automatic Urban and Rural Network (AURN). The annual concentration for each pollutant was calculated based on an average of hourly estimates within the grid cell containing the postcode centroid. The estimates were modelled for the year 2012 and linked to the participants’ postcode centroids at the baseline. The modelled results showed good performance when compared with monitoring data in the UK and the study postcodes. Based on monitoring sites around study postcodes, the root mean squared error (RMSE) was 3.12 for NO_2_ (*N* = 23), 5.54 for O_3_ (*N* = 13), 4.75 for PM_10_ (*N* = 19), and 1.20 for PM_2.5_ (*N* = 6). Full information is provided in Figure S1-S4 and Table S1 and descriptive statistics are reported in Table S2-S3.

### Covariates

Information on age, sex, and years of education was collected in the baseline interviews. Area deprivation and rural/urban categories were two measures at the Lower Layer Super Output Area level and were linked to participants using postcode information. Measures for area deprivation summarised different domains of area characteristics related to poverty and socioeconomic disadvantage including income, employment, education and training, health and disability, barriers to housing and services, living environment, and crime [27, 28]. The English Index of Multiple Deprivation 2010 and Welsh Index of Multiple Deprivation 2011 were used as these two versions corresponded to years of the baseline interviews. The English and Welsh versions use different components and methods but both scores indicate a ranking of deprivation in the regions. To generate a comparable measure for relative deprivation across the two cohort studies, the scores were divided into quintiles within England or Wales, with the highest quintile (Q5) for the most deprived areas. While air quality was one of indicators for the deprivation scores, their contributions were generally small (< 2%) and was unlikely to affect the quintiles. The 2011 Rural/Urban classification was used to indicate urban/rural settings in CFAS II and Wales [29]. An urban area was defined as physical settlements with a population of 10000 or more and all smaller settlements were classified as rural. Based on the density of settlements, further classification was applied in urban (major conurbation, minor conurbation, city and town) and rural areas (town and fringe, village, and hamlet and isolated dwelling).

### Analytical strategy

Descriptive statistics were carried out to investigate the distributions of exposure to the four air pollutants, individual and area level factors (rural/urban settings, area deprivation). Multistate hidden Markov modelling with continuous time [30] was used to examine the effects of air pollution exposure on risk of three transition states: from no dementia to dementia, from no dementia to death and from dementia to death (Figure S5). Due to variations in air pollution exposure across study centres, the four air pollutants were included as continuous variables but the interquartile range (IQR) scaled and quintile measures were also tested. The IQR-scaled measures allow comparison of different air pollutants and their effects on dementia. Since the IQRs in the overall populations were much larger than centre-specific values, it was more appropriate to scale by centre-specific IQRs. The quintile measures would largely overlap with study centres and it would be difficult to differentiate the effects of air pollution and study centres.

The unadjusted models were carried out for the four air pollutants and the adjusted models included age, sex, education, study centre, and area deprivation. Education, a known risk factor of dementia, was used to indicate individual socioeconomic positions. Area deprivation was used to indicate environmental factors related to air pollution and poor quality of living conditions (e.g., poor housing conditions, crime). Rural/urban categories were not included in the models as these categories partially overlapped with study centres and regional variation in air pollution exposure.

The analyses first focused on the overall population in CFAS II and Wales. Since the two cohorts were established at slightly different time points, the analyses were implemented separately. Given the large variation in air pollution exposure across the five study centres, further analyses were stratified by study centres. A random-effects meta-analysis was applied to summarise centre-specific estimates and calculate the pooled estimates since the two cohorts were carried out at different time points and contexts (e.g., urban/rural settings). The estimates of the five centres were added to nine longitudinal cohort studies reporting the impacts of four air pollutants on dementia [15, 16, 31–37]. The studies were identified from existing systematic reviews [6–10] and an updated literature search (Figure S6 and Table S8). The results were converted to per 1 µg/m^3^ increase in air pollution exposure and a random-effects meta-analysis was used to summarise estimates from all studies. The heterogeneity was measured by I-squared statistic (I^2^).

The multistate models were inverse probability weighted to account for study design and non-response rate at baseline as well as longitudinal attrition [38]. Sensitivity analyses based on different scenarios of those who did not participate the follow-up wave were carried out to ensure attrition did not affect the main results. To test the impacts of migration on the results, the 540 (4.8%) participants who reported that they had lived in their local areas for less than five years were excluded in the sensitivity analyses. More detailed information is provided in Table S5-S7. All analyses were carried out in R 4.1.2 and Stata 17.0.

## Results

Table 1 shows descriptive information on the baseline populations in CFAS II (*N* = 7753) and Wales (*N* = 3576). The median age was 75 (IQR = 12) years old in CFAS II and 74 (IQR = 11) in CFAS Wales. Both cohorts had more women than men and just over half had 10–11 years of education. The percentage of people with dementia was 5.9% in CFAS II and 5.5% in CFAS Wales. In CFAS II, 19% of the participants lived in the most deprived quintile while this figure was 15% in CFAS Wales. About 80% of the CFAS II participants lived in urban areas. In CFAS Wales, most participants (60%) lived in rural settings.

**Table 1:** Descriptive information on the study population at baseline (N (%))

	CFAS II	CFAS Wales
N	7753	3576
Age		
65–69	1939 (25.0)	1039 (29.1)
70–74	1872 (24.2)	915 (25.6)
75–79	1621 (20.9)	662 (18.5)
80–84	1275 (16.5)	499 (14.0)
85 +	1046 (13.5)	461 (12.9)
Sex		
Men	3529 (45.5)	1615 (45.2)
Women	4224 (54.5)	1961 (54.8)
Education		
≤ 9 years	2044 (26.8)	429 (12.2)
10–11 years	3921 (51.4)	1801 (51.1)
≥ 12 years	1667 (21.8)	1295 (36.7)
Missing	121	51
Dementia (baseline)		
No	7295 (94.1)	3379 (94.5)
Yes	458 (5.9)	197 (5.5)
Area deprivation		
Q1 (least)	1892 (24.4)	439 (12.3)
Q2	1590 (20.5)	827 (23.1)
Q3	1776 (22.9)	1074 (30.0)
Q4	1024 (13.2)	696 (19.5)
Q5 (Most)	1471 (19.0)	540 (15.1)
Rural/urban categories		
Urban major conurbation	2538 (32.7)	0 (0.0)
Urban minor conurbation	2559 (33.0)	0 (0.0)
Urban city and town	1041 (13.4)	1424 (39.8)
Rural town and fringe	1102 (14.2)	1028 (28.8)
Rural village	349 (4.5)	833 (23.3)
Rural hamlet & isolated dwelling	164 (2.1)	291 (8.1)

At the two-year follow-up wave, 5285 (68.2%) CFAS II participants were interviewed, 639 (8.2%) were dead before the interview and 1829 (23.6%) were lost to follow-up. For CFAS Wales, 2236 (62.5%) were interviewed, 193 (5.4%) were dead before the follow-up wave and 1147 (32.1%) were lost. The mean follow-up time of death was 60.7 months (SD = 17.6). More detailed information on transition states over time is provided in Table S4.

**Fig. 1:**
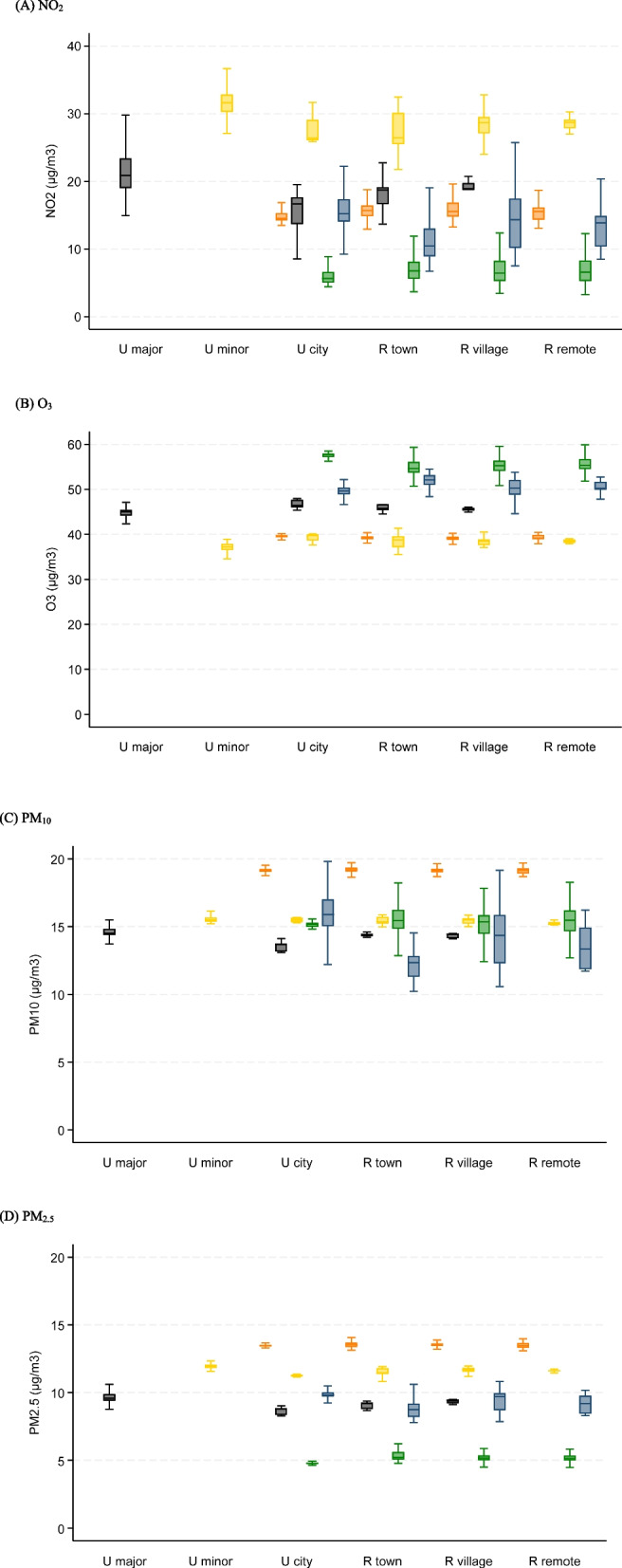
Box plots of air pollutants by study centre (Cambridgeshire – Orange; Newcastle upon Tyne – Black; Nottingham – Yellow; North Wales – Green; South Wales – Blue) and urban/rural settings (Urban major conurbation – U major; Urban minor conurbation – U minor; Urban city and town – U city; Rural town and fringe – R town; Rural village – R village; Rural hamlet and isolated dwelling – R remote)

The median exposure to NO_2_ was 21.1 µg/m^3^ (IQR = 14.2), 39.4 µg/m^3^ (IQR = 6.4) for O_3_, 15.5 µg/m^3^ (IQR = 4.2) for PM_10_, and 11.9 µg/m^3^ (IQR = 3.5) for PM_2.5_ among the two cohort participants (Table S2). Levels of air pollution exposure varied across the five study centres but remained similar across the six rural/urban categories in each centre (Fig. 1). There was a negative correlation between NO_2_ and O_3_ in the five centres while NO_2_, PM_10_, and PM_2.5_ were positively correlated (Table S3). More detailed information is provided in Table S2-S3.

The effects of the four air pollutants on dementia and death were generally small across the two cohorts (Table 2). After adjusting for age, sex, education, study centre and deprivation, a higher level of NO_2_ exposure was associated with higher risk of developing dementia (HR: 1.06; 95% CI: 1.01, 1.10) in CFAS II but not in the Welsh cohort (HR: 0.96; 95% CI: 0.88, 1.04). A higher level of O_3_ was associated with a lower risk of dementia (HR: 0.86; 95% CI: 0.77, 0.97) in CFAS II but a higher risk (HR: 1.16; 95% CI: 0.99, 1.37) in CFAS Wales. For PM_10_ and PM_2.5_, their effects sizes were similar to NO_2_ but they did not achieve statistical significance in either cohorts. The effects of air pollution exposure on transitioning to death were generally unclear.

**Table 2:** The effects of air pollutants (per 1 µg/m^3^ increase) on transition states of dementia and death

	NO_2_	O_3_	PM_10_	PM_2.5_
	HR (95% CI)	HR (95% CI)	HR (95% CI)	HR (95% CI)
**CFAS II**				
Model 1				
No to dementia	1.01 (0.99, 1.03)	1.00 (0.96, 1.04)	0.98 (0.92, 1.04)	0.97 (0.89, 1.05)
No to death	1.01 (1.00, 1.02)	1.00 (0.97, 1.02)	0.96 (0.92, 1.00)	0.97 (0.93, 1.02)
Dementia to death	1.01 (1.00, 1.02)	0.98 (0.96, 1.01)	1.00 (0.96, 1.05)	1.02 (0.97, 1.08)
Model 2				
No to dementia	1.06 (1.02, 1.11)	0.86 (0.76, 0.96)	1.10 (0.84, 1.44)	1.13 (0.74, 1.72)
No to death	1.00 (0.97, 1.03)	1.01 (0.94, 1.10)	1.01 (0.85, 1.20)	1.05 (0.81, 1.37)
Dementia to death	0.99 (0.96, 1.02)	1.02 (0.95, 1.09)	0.98 (0.82, 1.17)	0.98 (0.76, 1.28)
Model 3				
No to dementia	1.06 (1.01, 1.10)	0.86 (0.77, 0.97)	1.06 (0.80, 1.40)	1.06 (0.70, 1.62)
No to death	1.00 (0.97, 1.02)	1.01 (0.94, 1.09)	1.03 (0.88, 1.20)	1.10 (0.85, 1.41)
Dementia to death	0.99 (0.96, 1.02)	1.03 (0.95, 1.11)	0.95 (0.79, 1.14)	0.92 (0.70, 1.22)
**CFAS Wales**				
Model 1				
No to dementia	0.93 (0.90, 0.97)	1.11 (1.04, 1.18)	0.89 (0.79, 0.99)	0.92 (0.84, 1.00)
No to death	1.03 (1.01, 1.05)	0.94 (0.91, 0.98)	1.02 (0.96, 1.08)	1.06 (1.00, 1.12)
Dementia to death	0.96 (0.93, 0.99)	1.10 (1.05, 1.16)	1.17 (1.05, 1.30)	0.85 (0.79, 0.92)
Model 2				
No to dementia	0.87 (0.81, 0.93)	1.29 (1.16, 1.43)	0.89 (0.80, 0.99)	0.57 (0.41, 0.80)
No to death	1.04 (1.01, 1.06)	0.91 (0.86, 0.97)	1.01 (0.95, 1.07)	1.14 (0.95, 1.37)
Dementia to death	1.00 (0.96, 1.05)	1.11 (1.02, 1.22)	1.14 (1.02, 1.27)	0.95 (0.69, 1.30)
Model 3				
No to dementia	0.96 (0.88, 1.04)	1.16 (0.99, 1.37)	0.95 (0.82, 1.12)	0.82 (0.55, 1.23)
No to death	1.02 (0.99. 1.05)	0.94 (0.88, 0.99)	1.00 (0.93, 1.07)	1.07 (0.90, 1.28)
Dementia to death	1.04 (0.99, 1.08)	0.95 (0.87, 1.04)	1.04 (0.94, 1.14)	1.12 (0.87, 1.46)

Model 1: unadjusted

Model 2: adjusted for age, sex, study centre

Model 3: adjusted for age, sex, study centre, education, area deprivation

**Fig. 2:**
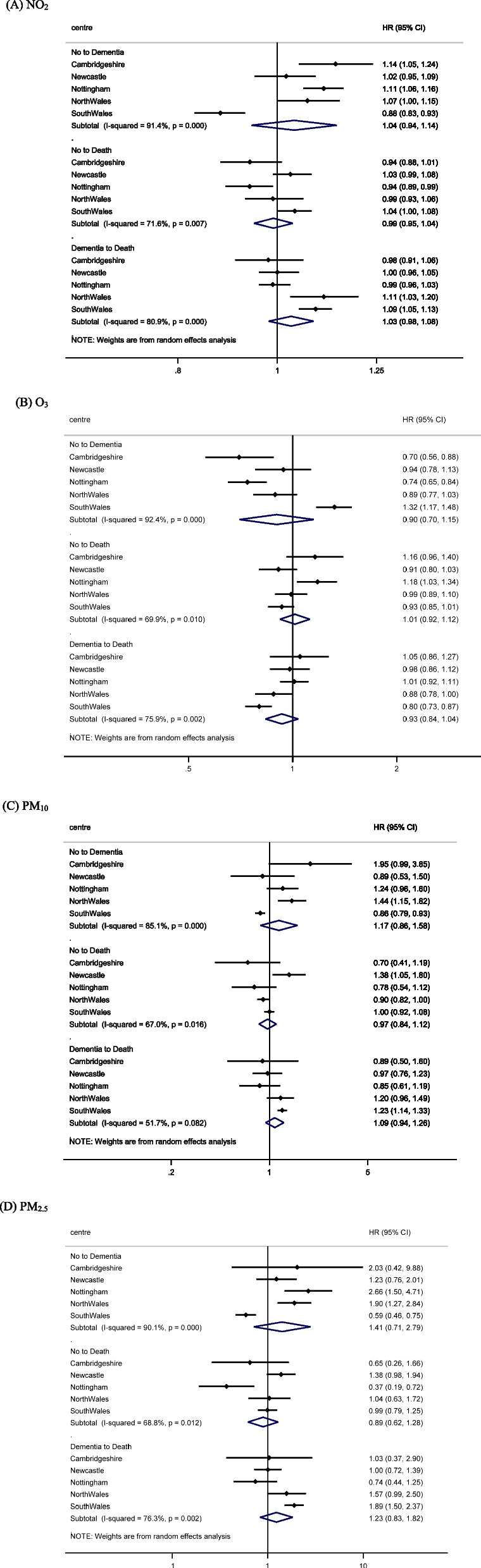
Associations between air pollution, dementia, and death by study centres (adjusted for age, sex, education, and area deprivation)

The results stratified by the five study centres are reported in Fig. 2. There was a lack of consistent trends across study centres with large heterogeneity (I^2^ = 51.7–92.4%). Based on the pooled estimates of five study centres, there was insufficient evidence suggesting the associations between NO_2_ (HR: 1.04; 95% CI: 0.94, 1.14), O_3_ (HR: 0.90; 95% CI: 0.70, 1.15), PM_10_ (HR: 1.17; 95% CI: 0.86, 1.58), PM_2.5_ (HR: 1.41; 95% CI: 0.71, 2.79) and increased risk of dementia. Similar patterns were also found for death. Air pollution had small effects on transition from no dementia and dementia to death.

**Fig. 3:**
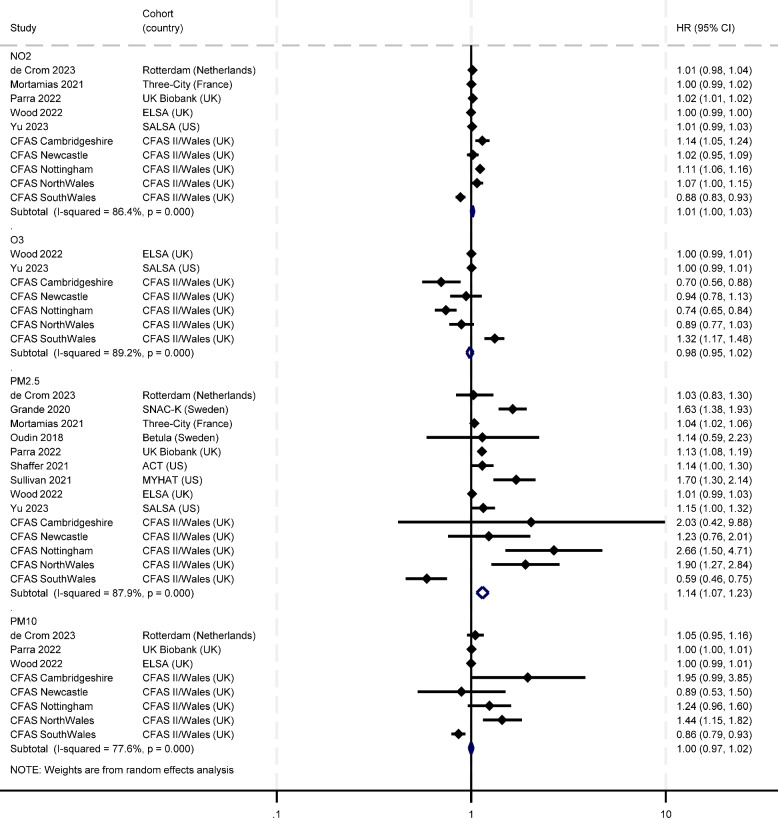
Meta-analysis of population-based cohort studies reporting the longitudinal associations between air pollution exposure and incident dementia (per 1 µg/m3 increase). Study [reference]: de Crom 2023 [31]; Grande 2020 [32]; Mortamias 2021 [33]; Oudin 2018 [34]; Parra 2022 [15]; Shaffer 2021 [35]; Sullivan 2021 [36]; Yu 2023 [37]; Wood 2022 [16]

Figure 3 reports meta-analyses of the current and previous cohort studies. Based on the pooled estimates, only PM_2.5_ showed an effect, with 1 µg/m^3^ increase being associated with a 14% higher risk of dementia (HR: 1.14; 95% CI: 1.07, 1.23). However, the heterogeneity was high (I^2^ = 87.9%). For the other air pollutants, the associations were close to null in NO_2_ (HR: 1.01; 95% CI: 1.00, 1.03), O_3_ (HR: 0.98; 95% CI: 0.95, 1.02), and PM_10_ (HR: 1.00; 95% CI: 0.97, 1.02). More detailed information on study designs and measurement methods is provided in Figure S6 and Table S8.

The results of sensitivity analyses are provided in Table S5-S7. Using the IQR-scaled and quintile measures did not alter the results (Table S5). The impacts of attrition on the results were found to be limited. The associations between air pollution and incident dementia became close to null when assuming a high percentage (50%) of dropouts had dementia at the follow-up wave (Table S6). The results remained similar after excluding the participants who had lived in the area for less than five years (Table S7).

## Discussion

Utilising two epidemiological cohort studies of older people in England and Wales and high-resolution modelled data on air pollutants, this study investigated the longitudinal associations between ambient air pollution exposure, dementia and death in later life. The effects of air pollution exposure on dementia were generally small, with inconsistent directions and strengths of associations observed across the two cohorts and five study centres.

### Strengths and limitations

CFAS II and Wales were designed to obtain representative samples of older people in the UK, including participants with different socioeconomic backgrounds and living in diverse settings. An algorithmic diagnosis method was used, allowing the same diagnostic standard to be applied to all the participants across different regions. The advanced air pollution model provided high resolution data, which could estimate exposure to outdoor air pollutants in the residential areas of the participants with less measurement error than standard models with coarser spatial scales. Multistate modelling was used to include all the participants with or without dementia at baseline and incorporate the competing risk outcome of death.

There were some limitations. The 2012 annual concentration of air pollutants was used to indicate the baseline exposure for the two cohort participants. These measures do not reflect cumulative exposure across the lifecourse and potential relocation of the participants over time. Yet most of the participants had lived in the area for at least five years before the baseline interview. Further excluding the participants with a short residential period (< 5 years) did not alter the results. People living in the same areas might expose to different levels of ambient air pollution due to travel behaviour, housing conditions and other environmental factors (e.g., green spaces) [39]. This study did not assess exposure to indoor air pollution sources (e.g., burning fuels for cooking and heating), which can be important for older people as they tend to spend more time indoors. However, adjustment for education and area deprivation may partially account for differences in housing and living conditions across individuals. This study did not control for lifestyle factors (e.g., physical activity, social engagement) and chronic conditions (e.g., diabetes, cardiovascular diseases) as they might be on the pathways between air pollution exposure and dementia. Although these effects could be tested in mediation analyses, this is beyond the scope of this study. The information on lifestyle factors and chronic conditions were collected in the CFAS II and Wales interviews but it was difficult to clarify their mediating roles as they were measured at the same time as the exposure. It can also be complicated to estimate direct and indirect effects in multistate modelling. The follow-up interviews of CFAS II and Wales were carried out two years after the baseline. It might be difficult to identify the long-term impacts of air pollution on the development of dementia over such a short time period. Participants who had poor health might have declined the interviews or died before the follow-up waves. To address this, inverse probability weights were applied to account for non-response at baseline and attrition over time. The sensitivity analyses further showed that the results remained similar even in some extreme scenarios.

### Interpretations of the results

The findings from CFAS II and Wales suggest limited effects of air pollution on dementia in later life. Our results were consistent with the findings from ELSA [16] but differed from the two previous UK-based studies which reported the negative impacts of PM_2.5_ and NO_2_ [14, 15]. All the three studies estimated exposure to air pollutants using annual concentration of the baseline years (2004 for ELSA and CPRD, 2010 for UK Biobank). Compared to previous studies using medical records and health administrative databases [13, 14, 40, 41], the meta-analysis of the current study and nine longitudinal cohort studies in Netherlands [31], Sweden [32, 34], France [33], US [35–37], and UK [15, 16], showed null associations for most air pollutants apart from PM_2.5_. The cohort studies were based on samples of the general population and used consistent assessment and diagnostic methods to identify dementia cases. Variations in research methods may lead to different conclusions as underdiagnosis, misdiagnosis and misclassification of dementia in the community settings may not be independent of air pollution exposure [9]. Different levels of air pollution across studies might also explain different findings. The same air pollution model [22] was applied to generate exposure estimates in ELSA [16], CPRD [14] and the current study. Compared to the estimated concentrations in Greater London and wider areas in England [14, 16], levels of ambient air pollution were generally lower in the study areas of CFAS II and Wales and, therefore, fewer participants lived in highly polluted environments.

This study did not observe consistent patterns across the five study centres. If exposure to air pollutants was assumed to have toxic and biological effects on the brain and cognition [42], dose response relationships or similar trends should be identified across regions and countries. While measures for air pollution exposure could be affected by several issues such as timing, measurement errors and exposure variability [9], regional variations might indicate that the modelled air pollution data were a proxy of other factors (e.g., access to services) [43]. Compared to dementia, the associations between air pollution and cognitive decline were less clear [11], even when measuring lifecouse exposure to air pollution [44]. Although air pollution is recognised as a key modifiable risk factor for dementia [11], the causal relationship and population level impact (i.e., addressing air pollution will reduce 2.3% worldwide dementia cases) might not be straightforward. Mechanisms between air pollution exposure, cognitive decline and dementia might be more complicated, involving interactions between individual factors and other community and environmental characteristics [45].

Opposite effects of NO_2_ and O_3_ on dementia were found in this study and the CPRD analyses [14]. Based on atmospheric chemistry, O_3_ is derived from reactions of nitrogen oxides (NO and NO_2_) and solar radiation and therefore they are negatively correlated during daylight hours in urban areas [46]. Yet, it has been suggested that both air pollutants have detrimental effects on cognitive health [6, 11]. This seems peculiar as there would be no relative risk when comparing people living in areas with high NO_2_ (low O_3_) vs high O_3_ (low NO_2_). Although several studies did not measure both NO_2_ and O_3_ at the same time, the literature could be influenced by publication bias where only statistically significant associations were submitted for review and published [47]. When focusing on the studies reporting the two air pollutants [13, 14, 40, 41], the effects of NO_2_ and O_3_ were not both detrimental. To clarify the role of these two air pollutants in brain health, it is important to capture exposure measures at the individual level and improve predicted values based on residential locations.

## Conclusions

This study suggests unclear associations between air pollution exposure and incident dementia across five areas of England and Wales. Incorporation of longitudinal population-based cohort studies in high-income countries shows some evidence for the detrimental effect of PM_2.5_ but not NO_2_, O_3_, and PM_10_. This seems different from the literature based on health administrative databases [13, 14]. To strengthen the evidence in this field, it is important to consider how methodological (e.g., sampling, measurement methods) and contextual factors (e.g., geographical areas, characteristics of study population) can affect the findings. Novel approaches are needed to better capture individual exposure levels of air pollution, including both indoor and outdoor sources [48, 49]. To estimate the long-term effects of air pollution on brain health, it is essential to clarify the timing of the exposure measurements in the lifecourse of the population. Measures for exposure levels, residential, lifestyle and health factors across the lifecourse would be required. Such data are not currently available in most cohort studies. Future research may also investigate interactions between air pollution, built and natural environmental factors and their complex impacts on cognitive health in older age [43]. It is essential to generate evidence from low- and middle-income countries [50], where dramatic changes in population structure, urbanisation and environmental pollution are occurring. This will provide a comprehensive approach to identify environmental risk factors for dementia and develop population-level interventions that support older people living in diverse settings across the world.

### Supplementary Information

**Supplementary Material 1.**

## Data Availability

The interview data from the Cognitive Function and Ageing Study II and Cognitive Function and Ageing Study Wales are available through the Dementia Platform UK (DPUK) data portal (https://portal.dementiasplatform.uk/CohortDirectory/Item?fingerPrintID=CFAS%20II, https://portal.dementiasplatform.uk/CohortDirectory/Item?fingerPrintID=CFAS%20Wales) and can be accessed by filling in an application form (https://portal.dementiasplatform.uk/Apply). The interview data from the Cognitive Function and Ageing Study Wales can also be accessed via UK Data Service (https://beta.ukdataservice.ac.uk/datacatalogue/studies/study?id=8281). The air pollution data are available from the corresponding author on reasonable request.
