# Supplementary material for: The longitudinal associations between ambient air pollution exposure and dementia in the UK: results from the cognitive function and ageing study II and Wales
Source: BMC Public Health. 2024 May 4;24:1233. doi: 10.1186/s12889-024-18723-3 (PMC11069162; doi:10.1186/s12889-024-18723-3)
Supplement: Supplementary file 1 — Supplementary Material 1. [file 12889_2024_18723_MOESM1_ESM.docx]

**The longitudinal associations between ambient air pollution exposure and dementia in the UK: results from the Cognitive Function and Ageing Study II and Wales**

**Supplementary File**

1. Modelled data for air pollutants 2012

2. Descriptive information on the four air pollutants

3. Transition states of multilevel modelling

4. Sensitivity analysis

4.1 IQR-scaled and quintile

4.2 Scenarios for attrition

4.3 Length of residence

5. Literature searches for air pollution and dementia

**Supplementary file 1. Modelled data for air pollutants 2012**

The annual mean concentrations of NO_2_, O_3_, PM_10_ and PM_2.5_ for 2012 have been predicted across Great Britain, with Figure S1 showing maps of NO_2_ concentrations for the cities where the postcode concentrations are needed. The maps show high concentrations of NO_2_ on major roads compared with smaller roads and the surrounding areas. The spatial gradient for O_3_ (not shown here) is opposite to NO_2_ due to the process of NO titration. The spatial gradient of PM_10_ and PM_2.5_ whilst similar to NO_2_ is smaller because of the significant contribution of regional PM to PM_2.5_ and PM_10_ mass.


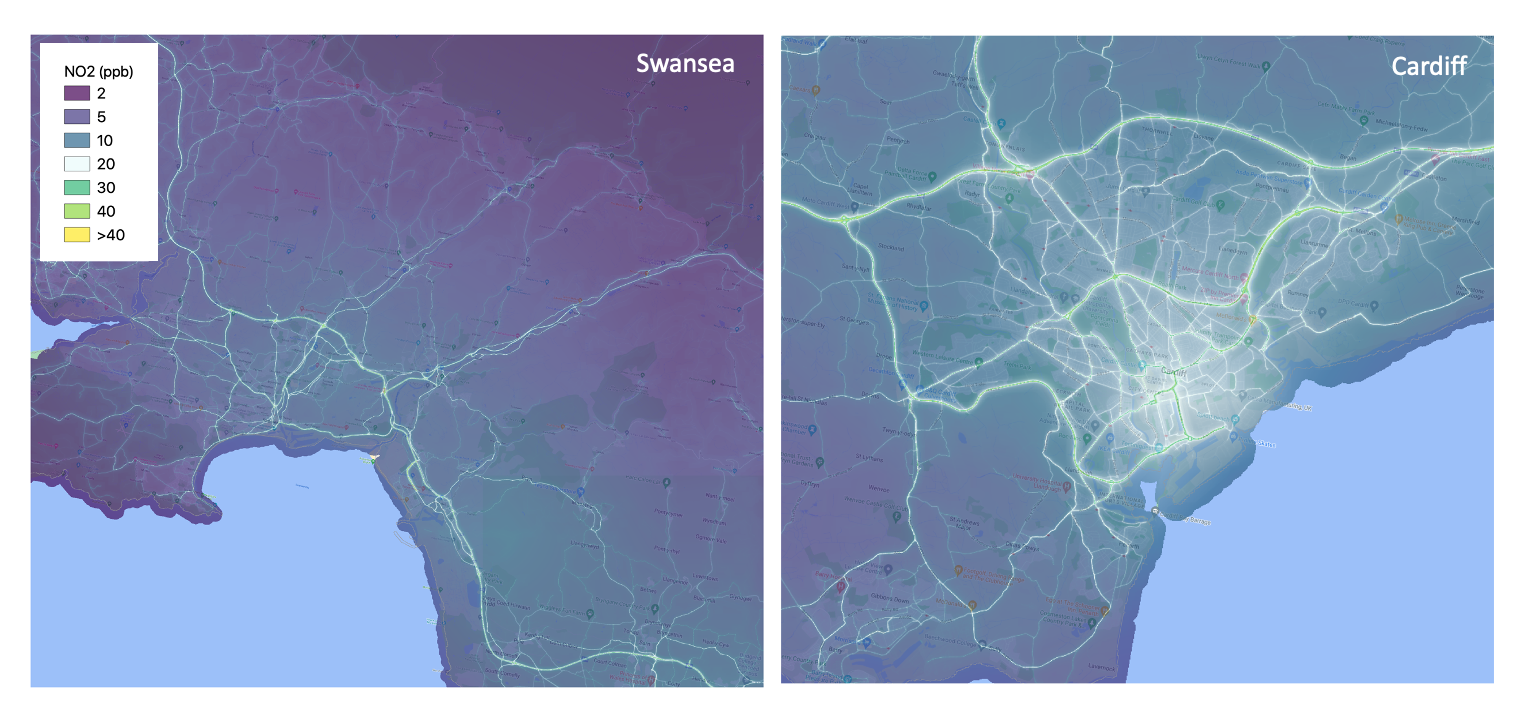


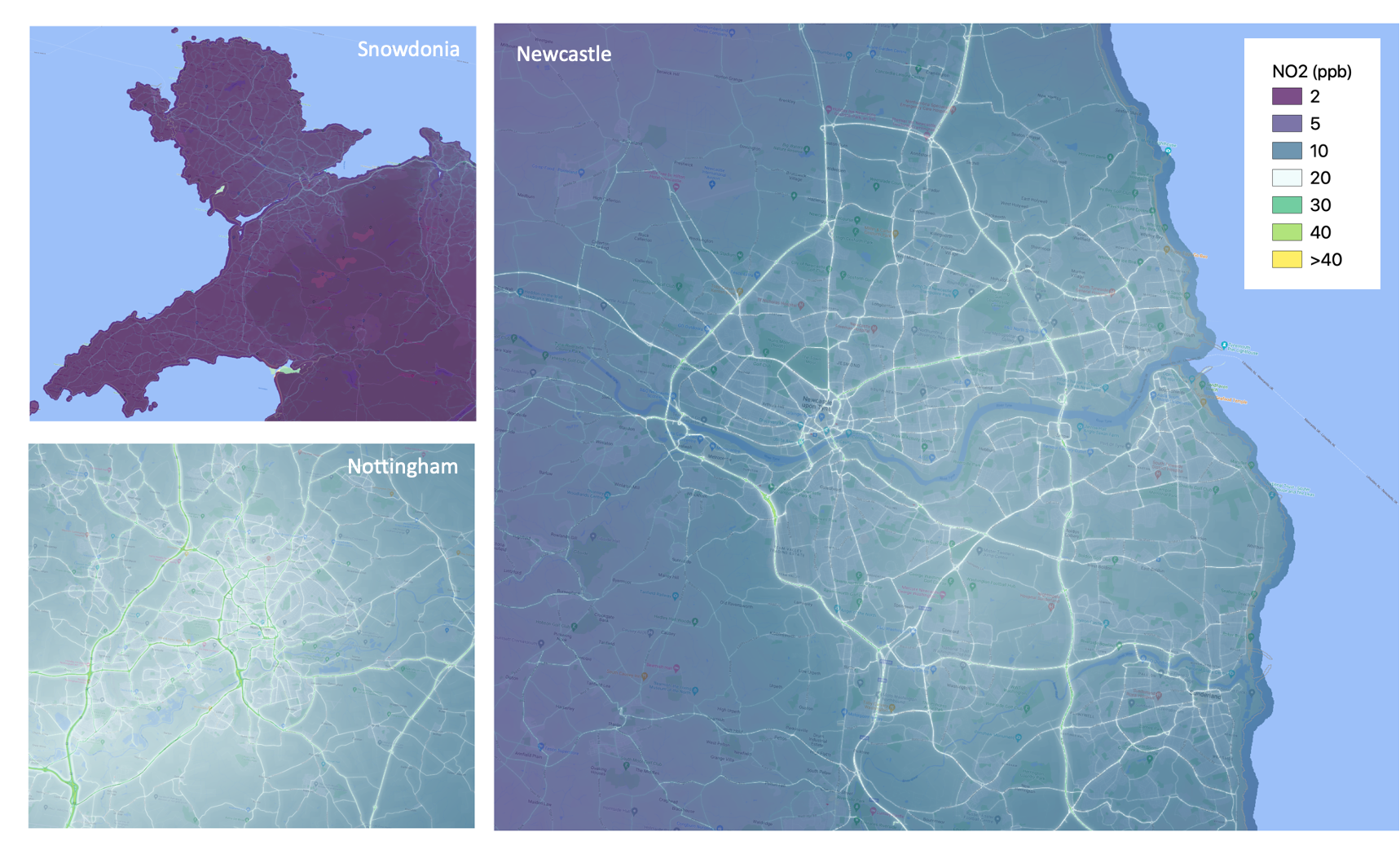


**Figure S1.** Annual mean NO_2_ concentrations for 2012 over Swansea, Cardiff, Snowdonia, Nottingham and Newcastle

**Comparison with measurements**

The modelled concentrations of O_3_, NO_2_, PM_2.5_ and PM_10_ for 2012 were assessed against the ground-based measurements from 371 sites across the UK (Figure S2). The data has been obtained from the Automatic Urban and Rural Network (AURN), London Air Quality Network (LAQN), Scotland Air Quality Network (SAQN), Wales Air Quality Network (WAQN), Air Quality England (AQE), and North Ireland Network (NI). The site categories include rural (36), urban background (90), suburban (16), roadside (186), kerbside (13), and industrial (30) sites.


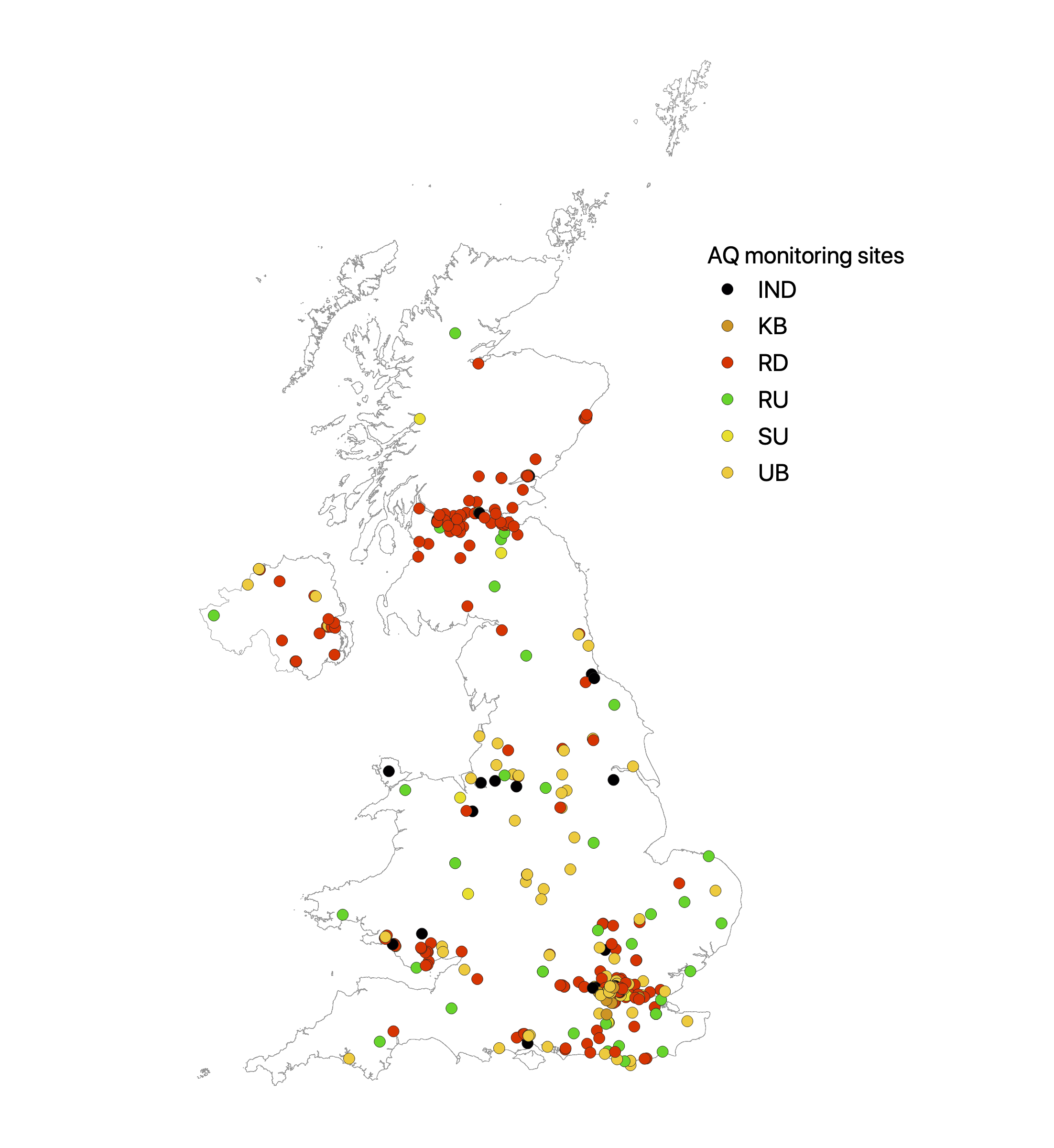


**Figure S2.** Air quality monitoring stations for model evaluation

Table S1 shows the performance statistics for all sites across the UK and at sites around the cohort’s postcodes. For UK, the statistics show large percentages of the predictions are within a factor of two of the measurements (FAC2 × 100), i.e., 93% for NO_2_, 100% for O_3_, 99% for PM_10_, and PM_2.5_. On average, the model slightly underestimates NO_2_ (-2.01 µg m^-3^ or -6%), O_3_ (-0.55 µg m^-3^ or -1%), PM_10_ (-2.02 µg m^-3^ or -10%), and PM_2.5_ (-0.82 µg m^-3^ or -6%). The RMSE and r values represent the model’s uncertainty, and its ability to describe the variation of NO_2_ (RMSE=13.67 µg m^-3^, r=0.76), O_3_ (RMSE=5.44 µg m^-3^, r=0.85), PM_10_ (RMSE=4.67 µg m^-3^, r=0.70) and PM_2.5_ (RMSE=2.83 µg m^-3^, r=0.57). All of the results show good model performance, although less so for PM_2.5_. The low r value for PM_2.5_ is likely to be driven by a number of outliers, particularly those in the city and/or near a train station, such as the Glasgow kerbside site (GLA4). In addition, the underprediction of PM_10_ and PM_2.5_ at very small industrial sites, such as the Anglesey Llynfaes site (ANG2) near a quarry, is partly responsible for the low r values.

In comparison with the statistics for all UK sites, the statistics for the sites close to the cohort postcodes show lower bias and better correlation with the measurements of NO_2_ and PM_2.5_. The negative bias in O_3_ is larger but only by a small margin (-2.72 µg m^-3^or -6%). The bias for PM_10_ is also smaller although the low r value is due to the underprediction at the industrial sites such as AGN2 site and Port Talbot Prince Street (MMF6) (circled in the PM_10_ scatter plot in Figure 5). The overprediction of PM_10_ at Swansea Morrison Roadside sites (SWA5) may also play role but this location is located over 2.5km away from the nearest postcode. Overall, whilst these results show good model performance, care should be taken when interpreting these statistics, which are based upon small numbers of monitoring sites.

**Table S1.** Performance statistics of NO_2_, O_3_, PM_10_ and PM_2.5_ predictions for 2012 for all sites in the UK (domain = UK) and just sites around postcodes (domain = postcodes)

| Domain | pollutant | Number of data | Observed mean  (µg m^-3^) | Modelled mean  (µg m^-3^) | FAC2 | MB  (µg m^-3^) | NMB | RMSE  (µg m^-3^) | r |
| --- | --- | --- | --- | --- | --- | --- | --- | --- | --- |
| UK | NO_2_ | 295 | 36.14 | 34.12 | 0.93 | -2.01 | -0.06 | 13.67 | 0.76 |
| UK | O_3_ | 119 | 43.07 | 42.52 | 1 | -0.55 | -0.01 | 5.44 | 0.85 |
| UK | PM_10_ | 225 | 20.07 | 18.05 | 0.99 | -2.02 | -0.10 | 4.67 | 0.70 |
| UK | PM_2.5_ | 79 | 13.14 | 12.33 | 0.99 | -0.82 | -0.06 | 2.83 | 0.57 |
| Postcodes | NO_2_ | 23 | 28.84 | 29.30 | 1.0 | 0.46 | 0.02 | 3.12 | 0.97 |
| Postcodes | O_3_ | 13 | 47.82 | 45.10 | 1.0 | -2.72 | -0.06 | 5.54 | 0.84 |
| Postcodes | PM_10_ | 19 | 19.59 | 18.58 | 0.95 | -1.01 | -0.05 | 4.75 | -0.21 |
| Postcodes | PM_2.5_ | 6 | 10.58 | 10.16 | 1.0 | -0.42 | -0.04 | 1.20 | 0.89 |

The box plots (Figure S3) indicate that the model can capture the rural-urban gradients of all pollutants well, particularly for NO_2_. The model tends to underestimate PM_10_ and PM_2.5_ at roadside and kerbside sites. The distribution of modelled PM_10_ and PM_2.5_ is however similar to the measurements at these sites, suggesting that the underprediction may be due to the underestimation of the emissions. The bias at industrial sites, particularly for PM_10_, may be due to the nature of uncertainty of emissions from these sources. The median value of modelled PM_10_ concentrations at rural sites agrees closely with the measurements although there is a smaller spread in concentrations. This may be driven by a large variety of PM_10_ sources that are hard to predict in the model, for instance, windblown dust.


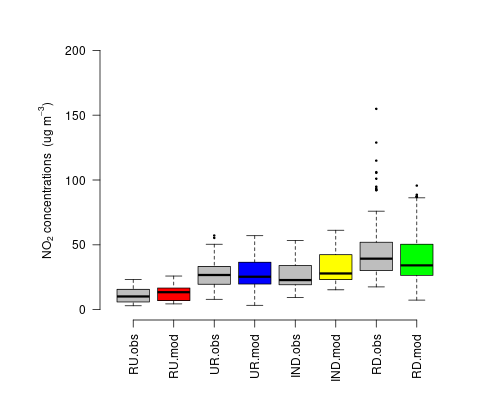

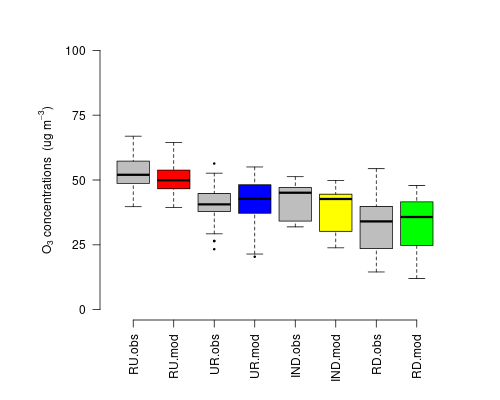


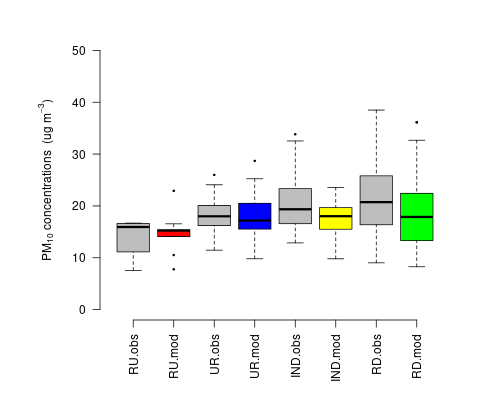

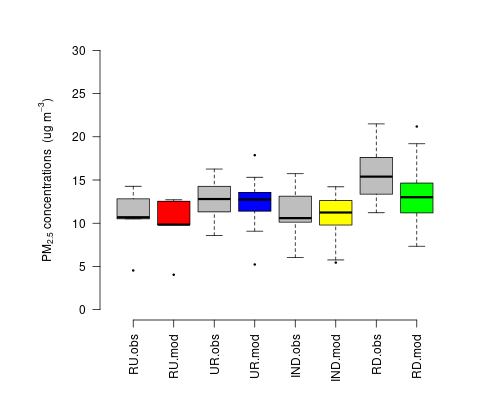


**Figure S3.** Spatial gradients of modelled and observed exposure to NO_2_, O_3_, PM_10_ and PM_2.5_ (RU = rural sites, UR = urban and suburban sites, IND = industrial sites, RD = roadside and kerbside sites)

Figure S4 shows the scatter plots of modelled and observed concentrations of NO_2_, O_3_, PM_10_ and PM_2.5_ for sites in or near Cambridge, Cardiff, Newcastle, Nottingham, and Snowdonia (purple), and the rest of the sites across the UK (yellow). The plots are consistent with the FAC2 values in the performance statistics.

The NO_2_ and O_3_ for the target towns are well within the factor of two of the measurements although PM_10_ and PM_2.5_ are more scattered than NO_2_ and O_3_. A large negative bias of PM_10_ predictions is visible at ANG2 (circled in red). This is likely to be due to CMAQ being unable to capture the impact of the local quarry close to this site. The footprint of PM from the quarry near AGN2 (estimated separately) does not reach the postcodes of interest, and its impact on local residents is therefore expected to be minimal. Overall, the majority of the sites within the boundaries of postcodes agree very well with the measurements.

**Figure S4.** Scatter plots of modelled and observed exposure to NO_2_, O_3_, PM_10_ and PM_2.5_ (RU = rural sites, SU = suburban sites, UB = urban background sites, IND = industrial sites, RD = roadside site, KB = kerbside sites). Purple points are sites within and near the coverage of targeted postcodes.

**Supplementary file 2. Descriptive information on the four air pollutants**

**Table S2.** Median and IQR of four air pollutants

|  | **NO_2_** | **O_3_** | **PM_10_** | **PM_2.5_** |
| --- | --- | --- | --- | --- |
| **CFAS II** | 21.1 (14.2) | 39.4 (6.4) | 15.5 (4.2) | 11.9 (3.5) |
| Cambridgeshire | 15.2 0(1.8) | 39.4 (0.7) | 19.2 (0.2) | 13.5 (0.2) |
| Newcastle | 20.8 0(4.3) | 45.0 (1.2) | 14.5 (0.4) | 9.6 (0.5) |
| Nottingham | 31.6 0(2.6) | 37.2 (1.4) | 15.5 (0.3) | 11.9 (0.2) |
| **CFAS Wales** | 9.9 0(8.5) | 53.0 (5.3) | 15.4 (2.0) | 5.8 (4.6) |
| North | 6.4 0(2.8) | 55.0 (2.5) | 15.4 (1.5) | 5.2 (0.5) |
| South | 14.8 0(4.2) | 49.9 (1.9) | 15.5 (2.9) | 9.8 (0.6) |

**Table S3.** Pearson correlation coefficients of the four air pollutants

| CFAS II | NO_2_ | O_3_ | PM_10_ | PM_2.5_ |
| --- | --- | --- | --- | --- |
| O_3_ | Total  -0.50 (-0.51, -0.48)  Cambridgeshire  -0.98 (-0.98, -0.98)  Newcastle  -0.98 (-0.98, -0.98)  Nottingham  -0.93 (-0.93, -0.92) | - | - | - |
| PM_10_ | Total  -0.50 (-0.52, -0.49)  Cambridgeshire  0.82 (0.81, 0.84)  Newcastle  0.85 (0.84, 0.86)  Nottingham  0.71 (0.69, 0.73) | Total  -0.45 (-0.47, -0.44)  Cambridgeshire  -0.82 (-0.83, -0.80)  Newcastle  -0.88 (-0.88, -0.87)  Nottingham  -0.76 (-0.77, -0.74) | - | - |
| PM_2.5_ | Total  -0.15 (-0.17, -0.13)  Cambridgeshire  0.68 (0.66, 0.70)  Newcastle  0.95 (0.95, 0.96)  Nottingham  0.92 (0.92, 0.93) | Total  -0.78 (-0.78, -0.77)  Cambridgeshire  -0.73 (-0.75, -0.71)  Newcastle  -0.96 (-0.96, -0.95)  Nottingham  -0.86 (-0.87, -0.84) | Total  0.90 (0.90, 0.91)  Cambridgeshire  0.87 (0.86, 0.88)  Newcastle  0.87 (0.86, 0.88)  Nottingham  0.80 (0.78, 0.81) | - |
| CFAS W | NO_2_ | O_3_ | PM_10_ | PM_2.5_ |
| O_3_ | Total  -0.92 (-0.93, -0.92)  North Wales  -0.71 (-0.73, -0.68)  South Wales  -0.92 (-0.92, -0.91) | - | - | - |
| PM_10_ | Total  0.40 (0.37, 0.42)  North Wales  0.36 (0.32, 0.40)  South Wales  0.78 (0.76, 0.80) | Total  -0.18 (-0.21, -0.15)  North Wales  0.05 (0.01, 0.10)  South Wales  -0.69 (-0.71, -0.66) | - | - |
| PM_2.5_ | Total  0.85 (0.84, 0.86)  North Wales  0.62 (0.60, 0.65)  South Wales  0.79 (0.77, 0.81) | Total  -0.90 (-0.91, -0.90)  North Wales  -0.79 (-0.81, -0.78)  South Wales  -0.77 (-0.79, -0.75) | Total  0.14 (0.11, 0.18)  North Wales  0.30 (0.26, 0.34)  South Wales  0.93 (0.92, 0.93) | - |

**Supplementary file 3. Transition states of multilevel modelling**

**Figure S5.** Transitions of no dementia, dementia and death in the multistate modelling

No dementia

Dementia

Death

**Table S4.** Numbers and percentages of transition states across five areas in CFAS II and Wales

| **CFAS II** |  |  | To |  |  |  |
| --- | --- | --- | --- | --- | --- | --- |
| Cambridgeshire | From | No | Dementia | Death | Censored | Total |
|  | No | 1667 (40.8) | 53 0(1.3) | 402 0(9.8) | 1966 (48.1) | 4088 |
|  | Dementia | 0 0(0.0) | 34 (15.2) | 114 (50.9) | 76 (33.9) | 224 |
| Newcastle | From |  |  |  |  |  |
|  | No | 1620 (40.3) | 640 (1.0) | 407 (10.1) | 1926 (47.9) | 4017 |
|  | Dementia | 0 0(0.0) | 45 (15.6) | 154 (53.3) | 90 (31.1) | 289 |
| Nottingham | From |  |  |  |  |  |
|  | No | 1701 (40.7) | 56 0(1.3) | 486 (11.6) | 1935 (46.3) | 4178 |
|  | Dementia | 0 0(0.0) | 22 (10.0) | 139 (63.5) | 58 (26.5) | 219 |
| **CFAS Wales** |  |  |  |  |  |  |
| North Wales | From |  |  |  |  |  |
|  | No | 1169 (39.9) | 270 (1.0) | 299 (10.2) | 1437 (40.9) | 2932 |
|  | Dementia | 0 0(0.0) | 61 (40.9) | 42 (28.2) | 46 (30.9) | 149 |
| South Wales | From |  |  |  |  |  |
|  | No | 955 (37.1) | 30 0(1.2) | 316 (12.3) | 1270 (49.4) | 2571 |
|  | Dementia | 0 0(0.0) | 136 (45.0) | 64 (21.2) | 102 (33.8) | 302 |

**Supplementary file 4. Sensitivity analysis**

**4.1 IQR-scaled and quintile**

**Table S5.** Hazard ratios of dementia and death per centre-specific interquartile range (IQR) and quintile increase in air pollutants (adjusted for age, sex, education, study centre and deprivation quintiles)

|  | IQR | Q2 vs Q1 | Q3 vs Q1 | Q4 vs Q1 | Q5 vs Q1 |
| --- | --- | --- | --- | --- | --- |
| **CFAS II** | HR (95% CI) | HR (95% CI) | HR (95% CI) | HR (95% CI) | HR (95% CI) |
| NO_2_ |  |  |  |  |  |
| No to dementia | 1.17 (1.05, 1.30) | 1.07 (0.63, 1.80) | 1.49 (0.79, 2.80) | 1.24 (0.57, 2.72) | 2.02 (0.79, 5.16) |
| No to death | 0.96 (0.89, 1.04) | 0.84 (0.62, 1.12) | 1.04 (0.72, 1.50) | 1.05 (0.68, 1.62) | 0.86 (0.52, 1.42) |
| Dementia to death | 0.96 (0.89, 1.04) | 0.98 (0.69, 1.40) | 0.84 (0.55, 1.29) | 0.96 (0.58, 1.58) | 0.93 (0.52, 3.68) |
| O_3_ |  |  |  |  |  |
| No to dementia | 0.92 (0.80, 1.04) | 0.51 (0.32, 0.81) | 0.34 (0.17, 0.69) | 0.55 (0.24, 1.25) | 0.66 (0.26, 1.68) |
| No to death | 1.01 (0.93, 1.09) | 1.30 (1.03, 1.64) | 1.67 (1.09, 2.54) | 1.60 (0.98, 2.62) | 1.10 (0.63, 1.92) |
| Dementia to death | 1.03 (0.95, 1.12) | 0.98 (0.74, 1.31) | 1.24 (0.78, 1.97) | 1.25 (0.74, 2.11) | 1.06 (0.58, 1.92) |
| PM_10_ |  |  |  |  |  |
| No to dementia | 1.00 (0.91, 1.09) | 1.06 (0.68, 1.66) | 0.79 (0.42, 1.49) | 1.40 (0.71, 2.75) | 0.80 (0.34, 1.87) |
| No to death | 1.00 (0.95, 1.05) | 1.35 (1.03, 1.76) | 1.42 (1.01, 2.03) | 1.06 (0.66, 1.70) | 1.22 (0.70, 2.13) |
| Dementia to death | 1.00 (0.94, 1.06) | 1.13 (0.85, 1.52) | 1.03 (0.68, 1.56) | 1.06 (0.68, 1.65) | 0.86 (0.49, 1.50) |
| PM_2.5_ |  |  |  |  |  |
| No to dementia | 1.00 (0.86, 1.15) | 0.85 (0.54, 1.34) | 1.14 (0.47, 2.77) | 1.68 (0.66, 4.26) | 1.13 (0.39, 3.26) |
| No to death | 1.00 (0.93, 1.08) | 1.35 (1.05, 1.75) | 0.99 (0.67, 1.46) | 0.93 (0.59, 1.46) | 0.94 (0.55, 1.60) |
| Dementia to death | 1.00 (0.92, 1.08) | 1.05 (0.79, 1.41) | 0.91 (0.56, 1.47) | 0.83 (0.49, 1.39) | 0.70 (0.38, 1.30) |
| **CFAS Wales** |  |  |  |  |  |
| NO_2_ |  |  |  |  |  |
| No to dementia | 1.00 (0.76, 1.31) | 1.37 (0.63, 2.98) | 1.31 (0.60, 2.87) | 1.44 (0.54, 3.88) | 0.39 (0.11, 1.38) |
| No to death | 1.07 (0.96, 1.20) | 1.17 (0.76, 1.80) | 1.22 (0.79, 1.87) | 0.97 (0.55, 1.71) | 1.62 (1.01, 2.62) |
| Dementia to death | 1.21 (1.04, 1.41) | 1.75 (0.97, 3.15) | 1.86 (1.01, 3.42) | 2.85 (1.29, 6.30) | 2.05 (0.93, 4.54) |
| O_3_ |  |  |  |  |  |
| No to dementia | 1.26 (0.80, 2.01) | 4.08 (1.32, 12.63) | 4.89 (1.43, 16.70) | 0.75 (0.13, 4.29) | 2.80 (0.65, 11.99) |
| No to death | 0.86 (0.75, 0.99) | 0.65 (0.44, 0.95) | 0.60 (0.34, 1.05) | 0.86 (0.44, 1.69) | 0.61 (0.29, 1.30) |
| Dementia to death | 0.87 (0.71, 1.07) | 1.35 (0.74, 2.46) | 1.21 (0.64, 2.28) | 0.94 (0.38, 2.32) | 0.80 (0.34, 1.86) |
| PM_10_ |  |  |  |  |  |
| No to dementia | 1.11 (0.82, 1.49) | 1.85 (0.98, 3.52) | 0.75 (0.32, 1.77) | 0.45 (0.18, 1.10) | 1.71 (0.91, 3.21) |
| No to death | 0.97 (0.84, 1.10) | 0.54 (0.34, 0.87) | 0.87 (0.63, 1.21) | 1.12 (0.83, 1.50) | 0.76 (0.52, 1.10) |
| Dementia to death | 1.15 (0.92, 1.44) | 1.83 (1.09, 3.07) | 0.89 (0.45, 1.77) | 0.81 (0.46, 1.44) | 1.72 (1.08, 2.72) |
| PM_2.5_ |  |  |  |  |  |
| No to dementia | 0.92 (0.73, 1.17) | 1.28 (0.48, 3.39) | 1.72 (0.58, 5.13) | 1.97 (0.50, 7.86) | 0.46 (0.10, 2.15) |
| No to death | 1.06 (0.95, 1.17) | 1.16 (0.71, 1.90) | 1.19 (0.65, 2.15) | 0.99 (0.44, 2.27) | 1.66 (0.80, 3.46) |
| Dementia to death | 1.09 (0.93, 1.27) | 1.21 (0.62, 2.35) | 1.22 (0.59, 2.54) | 2.06 (0.80, 5.32) | 1.08 (0.41, 2.84) |

**4.2 Scenarios for Attrition**

To reduce the complexity of modelling, the analyses focused on the associations between air pollution exposure and dementia in CFAS II using Poisson regression modelling (Matthews et al., 2016). The models were applied to the two cohort studies (adjusted for age, sex, deprivation quintiles, study centres) and the five study centres (adjusted for age, sex, and deprivation quintiles). Study weights were applied. Four approaches were carried out to test different scenarios:

(1) Crude (missing completely at random): The missing data and attrition were completely random. A Poisson regression was applied to data from 5161 participants without dementia at the baseline. The results were used as a reference.

(2) Imputed (missing at random): It was assumed that the loss to follow-up was related to some of the observed data. Multiple imputation was applied to those who were alive at wave 2 (N=6822) as people who died would not have a dementia diagnosis. Thirty imputed datasets were generated based on age, sex, education, study weights, self-rated health, disability, deprivation score and quintile, rural/urban categories, study centre, dementia (follow-up waves), MMSE scores (baseline and follow-up waves), care home status (baseline and follow-up waves), air pollution exposure (continuous variables and quintiles. Rubin’s rule was used to combine the results from the imputed datasets.

(3) Simulation 1 (missing not at random): It was assumed that attrition was due to dementia and people might have developed dementia and died in a short time period before the follow-up wave. To test this, a simple model was used to simulate dementia at wave 2 (N=7295). If a person was alive at the follow-up wave, a binomial distribution with a probability of 0.05 was applied (5% of people would have dementia at the follow-up wave). If a person was dead at the follow-up wave, a binomial distribution with a probability of 0.1 (10% of people with dementia) was applied. A follow-up year was imputed as a uniform distribution between 1 and 24. It was assumed that people who died at the follow-up wave had double the risk of developing dementia before death than those who were alive. Median values of the coefficients and 95% confidence intervals were reported based on 100 simulated datasets.

(4) Simulation 2: This was similar to Simulation 1. All participants who had missing dementia at the follow-up wave were imputed based on a binomial distribution of 0.5, which assumed that half of the dropouts (due to loss to follow-up or death) had dementia.

**Table S6.** Sensitivity analysis: association between air pollution and risk of developing dementia in CFAS II based on four scenarios

|  |  | Crude  (n=5161) | Multiple imputation  (n=6822) | Simulation 1  (n=7295) | Simulation 2  (n=7295) |
| --- | --- | --- | --- | --- | --- |
|  |  | IRR (95% CI) | IRR (95% CI) | IRR (95% CI) | IRR (95% CI) |
| NO_2_ | Overall | 1.06 (1.02, 1.10) | 1.06 (1.02, 1.10) | 1.04 (1.00, 1.08) | 1.01 (0.99, 1.03) |
|  | Cambridgeshire | 1.14 (1.05, 1.23) | 1.12 (1.03, 1.21) | 1.08 (1.01, 1.17) | 1.01 (0.97, 1.05) |
|  | Newcastle | 0.98 (0.90, 1.06) | 0.99 (0.92, 1.07) | 0.99 (0.93, 1.06) | 1.02 (0.98, 1.05) |
|  | Nottingham | 1.08 (1.05, 1.12) | 1.07 (1.03, 1.12) | 1.06 (1.02, 1.10) | 1.01 (0.98, 1.04) |
| O_3_ | Overall | 0.83 (074, 0.92) | 0.84 (0.75, 0.94) | 0.88 (0.80, 0.98) | 0.96 (0.91, 1.01) |
|  | Cambridgeshire | 0.71 (0.57, 0.88) | 0.74 (0.59, 0.92) | 0.80 (0.66, 0.98) | 0.96 (0.86, 1.08) |
|  | Newcastle | 1.05 (0.83, 1.32) | 1.01 (0.82, 1.25) | 1.01 (0.85, 1.21) | 0.94 (0.86, 1.03) |
|  | Nottingham | 0.78 (0.70, 0.87) | 0.81 (0.71, 0.93) | 0.84 (0.75, 0.95) | 0.98 (0.90, 1.06) |
| PM_10_ | Overall | 1.21 (0.94, 1.57) | 1.23 (0.96, 1.56) | 1.14 (0.91, 1.44) | 1.08 (0.96, 1.22) |
|  | Cambridgeshire | 1.91 (0.96, 3.77) | 1.86 (0.96, 3.62) | 1.51 (0.83, 2.70) | 1.09 (0.81, 1.48) |
|  | Newcastle | 0.80 (0.45, 1.42) | 0.89 (0.53, 1.48) | 0.94 (0.61, 1.41) | 1.13 (0.92, 1.39) |
|  | Nottingham | 1.32 (1.07, 1.64) | 1.35 (1.07, 1.69) | 1.23 (0.98, 1.54) | 1.00 (0.83, 1.21) |
| PM_2.5_ | Overall | 1.40 (0.84, 2.32) | 1.42 (0.86, 2.33) | 1.13 (0.93, 1.38) | 1.13 (0.93, 1.38) |
|  | Cambridgeshire | 2.39 (0.49, 11.64) | 2.55 (0.69, 9.49) | 1.22 (0.68, 2.16) | 1.22 (0.68, 2.16) |
|  | Newcastle | 0.92 (0.52, 1.61) | 1.00 (0.58, 1.74) | 1.17 (0.91, 1.49) | 1.17 (0.91, 1.49) |
|  | Nottingham | 2.33 (1.53, 3.55) | 2.18 (1.20, 3.94) | 1.01 (0.66, 1.54) | 1.01 (0.66, 1.54) |

**4.3 Length of residence**

**Table S7.** Sensitivity analysis: the associations between air pollutants, dementia and death after excluding the 540 participants who reported that they had lived in the area for less than five years (adjusted for age, sex, education and deprivation quintiles)

|  | NO_2_ | O_3_ | PM_10_ | PM_2.5_ |
| --- | --- | --- | --- | --- |
|  | HR (95% CI) | HR (95% CI) | HR (95% CI) | HR (95% CI) |
| **No to dementia** |  |  |  |  |
| Cambridgeshire | 1.11 (1.00, 1.23) | 0.78 (0.59, 1.02) | 1.45 (0.61, 3.44) | 1.09 (0.16, 7.50) |
| Newcastle | 0.99 (0.92, 1.07) | 0.99 (0.82, 1.21) | 0.72 (0.40, 1.29) | 1.10 (0.66, 1.81) |
| Nottingham | 1.14 (1.09, 1.20) | 0.70 (0.61, 0.80) | 1.25 (0.93, 1.67) | 3.27 (1.86, 5.77) |
| North Wales | 1.08 (1.01, 1.15) | 0.89 (0.78, 1.01) | 1.42 (1.12, 1.81) | 1.88 (1.26, 2.81) |
| South Wales | 0.90 (0.85, 0.94) | 1.26 (1.13, 1.40) | 0.88 (0.80, 0.94) | 0.61 (0.49, 0.77) |
| **No to death** |  |  |  |  |
| Cambridgeshire | 0.92 (0.85, 1.00) | 1.21 (0.99, 1.48) | 0.53 (0.28, 0.99) | 0.47 (0.17, 1.30) |
| Newcastle | 1.03 (0.99, 1.08) | 0.91 (0.80, 1.04) | 1.38 (1.05, 1.82) | 1.30 (0.92, 1.85) |
| Nottingham | 0.93 (0.88, 0.98) | 1.19 (1.05, 1.35) | 0.81 (0.57, 1.17) | 0.35 (0.18, 0.67) |
| North Wales | 0.99 (0.93, 1.06) | 0.99 (0.89, 1.11) | 0.93 (0.83, 1.03) | 1.13 (0.70, 1.82) |
| South Wales | 1.05 (1.02, 1.09) | 0.90 (0.82, 0.99) | 1.03 (0.95, 1.11) | 1.07 (0.86, 1.34) |
| **Dementia to death** |  |  |  |  |
| Cambridgeshire | 0.95 (0.87, 1.03) | 1.15 (0.91, 1.45) | 0.37 (0.32, 1.36) | 0.74 (0.21, 2.57) |
| Newcastle | 1.02 (0.97, 1.08) | 0.92 (0.80, 1.07) | 1.06 (0.75, 1.49) | 1.23 (0.83, 1.82) |
| Nottingham | 1.00 (0.97, 1.04) | 0.98 (0.89, 1.08) | 0.91 (0.65, 1.29) | 0.96 (0.58, 1.57) |
| North Wales | 1.12 (1.04, 1.20) | 0.89 (0.79, 0.99) | 1.22 (0.97, 1.52) | 1.57 (0.97, 2.55) |
| South Wales | 1.08 (1.05 1.12) | 0.81 (0.75, 0.88) | 1.22 (1.12, 1.32) | 1.85 (1.46, 2.34) |

**Supplementary file 5. Literature searches for air pollution and dementia**

A search of PubMed was carried out to identify systematic reviews/meta-analyses (systematic review OR meta-analysis OR metaanalysis OR meta analysis) related to air pollution (air pollution OR air pollutant OR air pollutants) and cognitive health (dementia OR cognitive function OR cognitive impairment) in older people (up to March 2023). The search identified 70 publications. Title screening was employed to exclude reviews focusing on children and specific subtypes of dementia (Alzheimer Disease or Vascular dementia only). This left 12 systematic reviews.

The cross-check of recent reviews^1-4^ found six longitudinal population-based cohort studies,^5-9,16^ which (1) included cohort studies of the general population or healthy volunteers; (2) applied consistent methods to identify dementia cases (3) reported the associations between any of the four air pollutants (NO_2_, O_3_, PM_10_, PM_2.5_) and dementia in this study. Using the search strategy in Weuve and colleagues (2021),^1^ an additional search was carried out in PubMed to identify new studies published from January 2021 to March 2023 (Figure S6). Three additional studies^10-12^ were found. Two studies based on cohorts of randomised controlled trials were excluded.^13,14^ One commentary was also excluded due to limited information.^15^

**Figure S6.** Diagram of literature research

Search of systematic reviews/meta-analyses

(N=55)

Relevant reviews

(N=12)

Longitudinal population-based cohort studies reporting results of NO_2_, O_3_, PM_10_, PM_2.5_ (N=6)

Additional search (01/2021-03/2023) using terms in Weuve et al., 2021 (N=360)

Longitudinal population-based cohort studies

(N=3)

Meta-analysis: longitudinal studies focusing on dementia (N=9)

**Table S8.** Summary of study designs, populations and results in longitudinal population-based cohort studies of air pollution and dementia

| Study | Study population |  |  | Measurement methods |  | Results | HR (95% CI) | | | |  |
| --- | --- | --- | --- | --- | --- | --- | --- | --- | --- | --- | --- |
|  | Location, year | Sample size (age range) | Data | Outcome: Dementia | Exposure: Air pollution (resolution) | Unit | NO_2_ | O_3_ | PM_2.5_ | PM_10_ | Adjustment |
| de Crom 2023 [10] | Rotterdam, Netherlands (2009-2018) | 7,511  (aged 55+) | The Rotterdam Study: population-based | Clinical diagnosis (MMSE, GMS, CAMDEX; DSM-III-R) | Land use regression from ESCAPE (1 x 1 km) | Per SD | 1.05  (0.95, 1.16) | - | 1.01  (0.93, 1.11) | 1.05  (0.95, 1.16) | Age, sex, smoking status, monthly household income, alcohol intake, physical activity, hours from home, body mass index, depressive symptoms |
| Grande 2020 [5] | Stockholm, Sweden (2001-2013) | 2,927  (aged 60+) | Swedish National Study on Aging and Care in Kungsholmen (SNAC-K): population-based | Clinical diagnosis (DSM-IV) | Gaussian dispersion model (60 x 60 m) | Per IQR | - | - | 1.54  (1.33, 1.78) | - | Age, sex, educational attainment, smoking, physical inactivity, socioeconomic status, early retirement, body mass index, depression, baseline MMSE score, cardiovascular risk factors |
| Mortamias 2021 [6] | Dijon, Bordeauz, Montpellier, France (1999-2013) | 7,066  (aged 65+) | Three-City Study: population-based | Clinical diagnosis (DSM-IV) | Land use regression from ELAPSE 2010 (100 x 100 m) and extrapolation for 1999-2012 (26 x 26 km) | Per 5 µg/m^3^ | 1.01  (0.95, 1.08) | - | 1.20  (1.08, 1.32) | - | Delayed entry with age, sex, centre, education APOE genotype, deprivation index, alcohol intake, smoking status |
| Oudin 2018 [7] | Umea, Sweden (1988-2014) | 1,806  (aged 55+) | Betula Study: population-based | Clinical diagnosis (DSM-IV) and medical records | Gaussian dispersion model (50 x 50m) | Per 1 µg/m^3^ | - | - | 1.14  (0.59, 2.23) | - | Age, sex, body mass index, waist-hip-ratio, alcohol, physical activity, smoking |
| Parra 2022 [16] | 22 sites in England, Scotland and Wales, UK (2006-2017) | 187,194  (aged 60+) | UK Biobank: healthy volunteers | Electronic health records (ICD 9/10) | Land use regression from ESCAPE (1 x 1 km) | Per IQR | 1.18  (1.10, 1.25) | - | 1.17  (1.10, 1.24) | 1.03  (0.98, 1.08) | Age, sex, college enrolled, smoking status, income, urban, research centre |
| Shaffer 2021 [8] | Puget Sound, Washington, US (1978-2018) | 4,166  (aged 65+) | The Adult Changes in Thought (ACT) study: population-based | Clinical diagnosis (CASI; DSM-IV) | Land use regression and geostatistical smoothing | Per 1 µg/m^3^ | - | - | 1.14  (1.00, 1.30) | - | Age, sex, educational degree, race, neighbourhood median household income |
| Sullivan 2021 [9] | Southwestern Pennsylvania, US (2006-2014) | 1,572  (aged 65+) | The Monongahela Youghiogheny Healthy Ageing Team (MYHAT): population-based | Clinical Dementia Rating (CDR) score | Community Multiscale Air Quality Model (12 x 12 km) | Per 1 µg/m^3^ | - | - | 1.70  (1.30, 2.14) | - | Age, sex, education, smoking |
| Wood 2022 [12] | England, UK (2004-2017) | 8,525  (aged 50+) | ELSA: population-based | Self-reported dementia (diagnosed by a doctor) | Community Multiscale Air Quality Urban model (20 x 20 m) | Per 10 µg/m^3^ | 0.97  (0.89, 1.05) | 1.01  (0.94, 1.09) | 1.10  (0.88, 1.37) | 0.98  (0.89, 1.08) | Age, sex, physical activity, smoking status |
| Yu 2023 [11] | Sacramento Valley, US (1998-2007) | 1,612  (aged 60+) | The Sacramento Area Latino Study on Ageing (SALSA): population-based study of Latin American | Clinical diagnosis (3MSE, SEVLT; DSM-IV) | Land use regression (30 x 30 m) | Per IQR | 1.14  (0.86, 1.49) | 1.07  (0.86, 1.34) | 1.33  (1.00, 1.76) | - | Age, sex, education, longest held occupation, neighbourhood SES, living county, outdoor physical activity, smoking status, household income |
| Current study | England: Cambridgeshire, Newcastle upon Tyne, Nottingham (2008-2013)  Wales: Gwynedd /Ynys Môn, Neath Port Talbot (2011-2016), UK | 11,329  (aged 65+) | CFAS II and Wales: population-based | Algorithmic diagnosis based on cognitive tests (GMS-AGECAT; DSM-III-R) | Community Multiscale Air Quality Urban model (20 x 20 m) | Per 1 µg/m^3^ | 1.04  (0.94, 1.14) | 0.90  (0.70, 1.15) | 1.41  (0.71, 2.79) | 1.17  (0.86, 1.58) | Age, sex, education, area deprivation, stratified by study centres |
|  |  |  |  |  |  |  |  |  |  |  |  |
| Pooled estimate | |  |  |  |  | Per 1 µg/m^3^ | 1.01  (1.00, 1.02) | 1.00  (1.00, 1.01) | 1.14  (1.07, 1.22) | 1.00  (1.00, 1.01) |  |
| Heterogeneity (I^2^) | |  |  |  |  |  | 68.4 | 0.0 | 86.7 | 0.0 |  |
| Pooled estimate  (Excluded UK biobank) | |  |  |  |  | Per 1 µg/m^3^ | 1.00  (0.99, 1.01) |  | 1.14  (1.07, 1.22) | 1.00  (0.99, 1.01) |  |
| Heterogeneity (I^2^) | |  |  |  |  |  | 0.0 |  | 85.3 | 1.5 |  |

**References**

[1] Weuve J, Bennett EE, Ranker L, et al. Exposure to air pollution in relation to risk of dementia and related outcomes: an updated systematic review of the epidemiological literature. Environ Health Perspect. 2021;129(9):96001.

[2] Zhao YL, Qu Y, Ou YN, Zhang YR, Tan L, Yu JT. Environmental factors and risks of cognitive impairment and dementia: A systematic review and meta-analysis. Ageing Res Rev. 2021;72:101504. doi:10.1016/j.arr.2021.101504.

[3] Wilker EH, Osman M, Weisskopf MG. Ambient air pollution and clinical dementia: systematic review and meta-analysis. BMJ. 2023;381:e071620. doi:10.1136/bmj-2022-071620

[4] Abolhasani E, Hachinski V, Ghazaleh N, Azarpazhooh MR, Mokhber N, Martin J. Air pollution and incidence of dementia: a systematic review and meta-analysis. Neurology. 2023;100(2):e242-e254. doi:10.1212/WNL.0000000000201419

[5] Grande G, Ljungman PLS, Eneroth K, Bellander T, Rizzuto D. Association Between Cardiovascular Disease and Long-term Exposure to Air Pollution With the Risk of Dementia. JAMA Neurol. 2020;77(7):801-809. doi:10.1001/jamaneurol.2019.4914

[6] Mortamais M, Gutierrez LA, de Hoogh K, et al. Long-term exposure to ambient air pollution and risk of dementia: Results of the prospective Three-City Study. Environ Int. 2021;148:106376. doi:10.1016/j.envint.2020.106376

[7] Oudin A, Segersson D, Adolfsson R, Forsberg B. Association between air pollution from residential wood burning and dementia incidence in a longitudinal study in Northern Sweden. PloS One 2018;13:e0198283–e0198283.

[8] Shaffer RM, Blanco MN, Li G, et al. Fine Particulate Matter and Dementia Incidence in the Adult Changes in Thought Study. Environ Health Perspect. 2021;129(8):87001. doi:10.1289/EHP9018

[9] Sullivan KJ, Ran X, Wu F, et al. Ambient fine particulate matter exposure and incident mild cognitive impairment and dementia. J Am Geriatr Soc. 2021;69(8):2185-2194. doi:10.1111/jgs.17188

[10] de Crom TOE, Ginos BNR, Oudin A, Ikram MK, Voortman T, Ikram MA. Air Pollution and the Risk of Dementia: The Rotterdam Study. J Alzheimers Dis. 2023;91(2):603-613. doi:10.3233/JAD-220804

[11] Yu Y, Haan M, Paul KC, et al. Metabolic dysfunction modifies the influence of traffic-related air pollution and noise exposure on late-life dementia and cognitive impairment: A cohort study of older Mexican-Americans. Environ Epidemiol. 2020;4(6):e122. doi:10.1097/EE9.0000000000000122

[12] Wood D, Evangelopoulos D, Beevers S, Kitwiroon N, Katsouyanni K. Exposure to ambient air pollution and the incidence of dementia in the elderly of England: the ELSA cohort. Int J Environ Res Public Health. 2022;19(23):15889. doi:10.3390/ijerph192315889

[13] Semmens EO, Leary CS, Fitzpatrick AL, et al. Air pollution and dementia in older adults in the Ginkgo Evaluation of Memory Study. Alzheimers Dement. 2023;19(2):549-559. doi:10.1002/alz.12654

[14] Wang X, Younan D, Millstein J, et al. Association of improved air quality with lower dementia risk in older women. Proc Natl Acad Sci U S A. 2022;119(2):e2107833119. doi:10.1073/pnas.2107833119

[15] Åström DO, Adolfsson R, Segersson D, Forsberg B, Oudin A. Local Contrasts in Concentration of Ambient Particulate Air Pollution (PM2.5) and Incidence of Alzheimer's Disease and Dementia: Results from the Betula Cohort in Northern Sweden. J Alzheimers Dis. 2021;81(1):83-85. doi:10.3233/JAD-201538

[16] Parra KL, Alexander GE, Raichlen DA, Klimentidis YC, Furlong MA. Exposure to air pollution and risk of incident dementia in the UK Biobank. Environ Res. 2022;209:112895. doi:10.1016/j.envres.2022.112895.
